# Placenta abruption in a woman with Wilson’s disease: a case report

**DOI:** 10.4076/1757-1626-2-8699

**Published:** 2009-08-07

**Authors:** Theodoros D Theodoridis, Leonidas Zepiridis, Dimitrios Athanatos, Konstantinos Dinas, Filippos Tzevelekis, John N Bontis

**Affiliations:** 1 First Department of Obstetrics and Gynaecology, Aristotle University of Thessaloniki, “Papageorgiou” General HospitalRing Road, N. Efkarpia, 56403, ThessalonikiGreece; 2 Second Department of Obstetrics and Gynaecology, Aristotle University of Thessaloniki, “Ippokration” HospitalGreece

## Abstract

Wilson’s disease is a rare genetic disorder of copper metabolism that causes primary hepatic cirrhosis, secondary menstrual abnormalities and infertility. Following the appropriate therapy patients are asymptomatic and pregnancy may be achieved. We present a case of placental abruption in a pregnant woman with Wilson’s disease and we review the management dilemmas and treatment options of pregnant women with Wilson’s disease.

## Introduction

Wilson’s disease is an inherited autosomal recessive disorder of copper metabolism caused by defective copper transporting ATPase in the liver. Because of impaired biliary secretion, copper remains in the liver, resulting in chronic hepatitis, hepatic cirrhosis and central nervous system dysfunction [1]. Wilson’s disease is a rare disorder with a worldwide prevalence of 1:30.000 [2]. Presentation is more common in the early twenties. With appropriate chelating therapy patients are expected to be asymptomatic, when therapy begins very early or when patients are diagnosed in an asymptomatic stage of the disease. Prior to introduction of proper medical treatment, successful pregnancies were rare due to reduced fertility (menstrual irregularities and early onset of chronic liver disease) [3]. However, when early diagnosis is made and proper therapy is administrated, pregnancy can be achieved.

## Case report

A 28-year old Caucasian woman (gravida 1, para 0) was referred to our outpatient antenatal clinic at the 6^th^ week of her pregnancy for her booking appointment. She had been trying to conceive for the previous 4 years. She reported that she was suffering from Wilson’s disease since puberty and she had been on penicillamine 1 g/day for more than 10 years. At the time she got pregnant she was completely asymptomatic.

Proper counseling was offered concerning all the possible implications of her pregnancy and the need of continuation of her chelating medical treatment. A low dose regimen of penicillamine 500 mg/day was commenced, according to the hepatologist advice. She attended all the routine antenatal appointments regularly. All her blood tests were within normal range, particularly those indicating liver function. Folic acid supplementation was prescribed during the first trimester in a daily dose of 400 μg, while no iron supplementation was needed throughout her pregnancy. The fetal nuchal translucency was measured 1,1 mm at 12 weeks of pregnancy, while the fetal anomaly scan at 22 weeks showed no obvious abnormality. Another two scans were performed antenatally in 28 and 32 weeks of gestation, in order to check the fetal growth and well-being.

She was admitted at 37 weeks in the labour ward with antepartum haemorrhage and constant abdominal pain, which started 1 hour prior to her admission. An ultrasound scan was performed and a large retroplacental haematoma was detected suggestive of placenta abruption. An emergency uncomplicated caesarean section was performed and a male infant weighed 2870 g was delivered. The ultrasound finding of placenta abruption was confirmed intraoperatively. The fetal APGAR score was 8 and 9 at the 1^st^ and 5^th^ minute respectively and did not need any kind of resuscitation. Her post-operative course was uneventful and she was discharged home with her baby on the 4^th^ postoperative day. Her postnatal obstetric and medical appointments revealed no obvious pathology. Shortly postnatally her hepatologist recommenced her with 1 gr/day of penicillamine as before pregnancy.

## Discussion

Wilson’s disease is a genetic disorder in which there is excessive accumulation of copper in the liver and brain because of an inherited defect in its biliary excretion [4]. It is transmitted from generation to generation by autosomal recessive inheritance. Homozygote of the specific mutations of both alleles of the Wilson’s disease gene or doubly heterozygous for different gene mutations may manifest the full clinical course of the disease [1].

Responsible for the transport of copper from the hepatocytes to bile is the protein ATP7B. The Wilson’s disease gene mutation leads to absence or diminished function of ATP7B, resulting in a decrease in biliary copper excretion [5].

The copper excess accumulates progressively in the liver causing toxic changes. When the capacity of the liver is exceeded, copper diffuses into the blood stream and distributes to other organs, mainly the brain, kidneys and eyes [1]. Clinical manifestations are rare before adolescence, but eventually develop in all untreated patients by early twenties [6]. Presentation varies depending on the organ mostly affected by the abnormal copper accumulation, and can be with hepatic dysfunction when liver is affected and neurologic disturbances and Kayser - Fleischer rings in the cornea when copper deposition in the brain or eyes is excessive.

There are two major issues in the reproductive profile of women with Wilson’s disease: infertility due to menstrual irregularities and recurrent miscarriages at the first trimester. The former is the result of hormonal changes caused by hepatic failure and copper toxicity [3] and the later is caused probably because of increased copper deposition in the uterus [7].

These two factors, including chronic liver disease, endocrinal disorders and anaemias, can explain why there are only few case reports in the literature about successful pregnancies in women with Wilson’s disease. Despite these facts it seems that patients who receive proper treatment may be able to conceive and reach a favorable outcome of pregnancy [1,4,8].

Currently there is four drugs used as anticopper agents in non-pregnant women: penicillamine, zinc, trientine and tetrathiomolybdate [1]. They all show about the same effectiveness, but their usage during pregnancy is always a matter of debate in relation to possible teratogenicity.

Penicillamine is the most widely used medication. Since the first documentation of its effectiveness [9], penicillamine has been regularly used to keep pregnant women asymptomatic. The dosage of 500 mg/day proves to be almost harmless to the fetus and quite effective at the same time for the treatment of the disease [5,7,8], although the dosage depends basically on the individual data of the patient and the stage of the copper-intoxication. Despite these studies, proper counseling should always be offered to pregnant women and their families regarding birth defects caused by penicillamine in larger doses, such as cutis laxa like syndrome, micrognathia, low-set ears, hyperflexibility of joints, fragile veins, varicosity and impaired wound healing [10]. Close monitoring of women throughout their pregnancies is crucial in order to prevent early symptoms of the disease due to inadequate therapy and to check their compliance with treatment.

Zinc is the second most used regiment for Wilson’s disease. Although the evidence is scant, the existing literature suggests that it is equally harmless and effective to penicillamine for the treatment of pregnant women [11]. Unfortunately there are no data in the literature concerning the usage of the other two agents during pregnancy.

The optimal mode of delivery varies according to the physical status of the pregnant woman. In healthy asymptomatic women vaginal delivery should be the first choice, while caesarean section should be reserved for emergencies or when other pathology is present. In our case placental abruption was the indication for performing a caesarean section. It should also be noted that to the best to our knowledge this is the first case in the literature of placenta abruption in a pregnant woman with Wilson’s disease. We believe that copper deposition in uterus may has caused the abruption of the placenta, since we could not detect any of the known causes or predisposing factors for placenta abruption. We must also state that this hypothesis is only suggestive, as there are no data of the level of copper in the woman’s blood at the time of ceasarean section or placental histology.

## Conclusion

It is clear that the reproductive status of women with Wilson’s disease is quite problematic. With proper medical treatment and close monitoring before and during pregnancy, a successful outcome can be achieved.
